# Safe thyroidectomy with intraoperative methylene blue spraying

**DOI:** 10.1186/1756-6614-5-15

**Published:** 2012-11-13

**Authors:** Serkan Sari, Erhan Aysan, Mahmut Muslumanoglu, Yeliz E Ersoy, Huseyin Bektasoglu, Erkan Yardimci

**Affiliations:** 1 Deparment General Surgery, Bezmialem Vakif University, Faculty of Medicine, Vatan Caddesi, Fatih, Istanbul, Turkey; 2 Department General Surgery, Istanbul Training and Research Hospital, Fatih, Istanbul, Turkey

**Keywords:** Methylene blue, Thyroid, Safe, Intraoperative, Spraying

## Abstract

**Background:**

We aimed to minimalize operative complications by spraying of methylene blue stain on thyroid glands and the perithyroidal area.

**Material and methods:**

The intra-operative methylene blue spraying technique was used prospectively on a total of 56 patients who had undergone primary (not recurrent) thyroid surgery for a variety of thyroid diseases. Bilateral total thyroidectomy was performed in all cases. After superior but before inferior pole ligation, 0.5ml of methylene blue was sprayed over the thyroid lobe and perilober area. Tissues, especially parathyroides, the recurrent laryngeal nerve, and the inferior thyroid artery, were identified and evaluated.

**Results:**

Recurrent laryngeal nerve and arteries were not stained and thus they remained white in all cases while all other tissues were stained blue. Within three minutes parathyroid glands washed out the blue stain and the original yellow color was regained. Thyroid tissue wash-out time was not less than 15 minutes; perithyroideal muscles, tendinous and lipoid structures took no less than 25 minutes.

**Conclusion:**

The safety of intravascular methylene blue guidance on thyroid surgery is known. This research demonstrates the effectiveness of the spraying technique, a new technique which ensures not only identification of parathyroid glands within three minutes, but also identification of recurrent laryngeal nerves and inferior thyroid arteries.

## Introduction

Disorders of the thyroid gland constitute the second most common endocrine disease following diabetes mellitus [1]. Thyroidectomy is one of the most frequent operations performed in iodine-deficient regions [2,3]. The main postoperative complications of thyroidectomy are recurrent laryngeal nerve (RLN) palsy and hypoparathyroidism [4-6].

Postoperative hypocalcemia after thyroidectomy leads to patient discomfort and prolonged hospital stay. The etiology appears to be multifactorial, i.e. iatrogenic hypoparathyroidism, the extent of surgery, the number of functioning glands remaining and the surgeon’s experience [7-11].

Although the overall incidence of RLN palsy is low, when it does occur nerve palsy is a devastating life-long handicap. Anatomic identification of the RLN has long been accepted as the safest way of reducing nerve injury rates.

Selective in-vivo staining of the parathyroid glands by intravenous and intra-arterial administration of toluidine blue was first described in dogs by Klopper et al. in 1966 [12]. The withdrawal of toluidine blue from general use because of its negative side effects led to a search for an alternative dyestuff.

In the present study, we aimed to investigate whether methylene blue spraying technique during bilateral total thyroidectomy allowed us to safely identify RLN and parathyroid glands.

## Material and methods

The study was approved by the local ethics committee, registered to clinical trial (ID: NCT01347606). Informed consent was obtained from all patients.

Between January 2010 and January 2011, 56 consecutive patients with benign and malignant goiter disorders underwent total or near total thyroidectomy at the Bezmialem Vakif University Hospital and the Istanbul Training and Research Hospital. Indications for surgical treatment were as follows: multinodular goiter (n=38), solitar adenoma (suspicious cytology, n=11), and thyroid carcinoma (n=7). Exclusion criteria were as follows: reoperative surgery, presence of preoperative cord dysfunction, giant goiter, Grave’s disease, and retrosternal goiter.

Operations were performed by experienced endocrine surgeons using general anesthesia. In each patient, both before and after surgery indirect laryngoscopic examination was performed to evaluate vocal cord motility.

Hypocalcemia was defined as a postoperation serum calcium level of <8 mg/dL. The presence of clinical symptoms or signs of hypocalcemia were noted.

After a standard Kocher incision, bilateral total thyroidectomy was performed. Thyroideal lobe dissections were started when lateral thyroideal vein ligation was available. The superior pole was ligated and cut. At this point in the surgery, neither the parathyroid glands nor RLN can be easily identified.

Before inferior pole ligation, the thyroid lobe was deviated medially and 0.5ml methylene blue (BucoBleu®, 15ml ampul, Sandoz Co. Turkey) was sprayed over the thyroid lobe and perilober area. This area includes the parathyroides, inferior thyroid artery, veins, recurrent laryngeal nerve and perithyroidal muscles, and tendinous and lipoid structures. Tissues were identified and evaluated, especially the parathyroides and recurrent laryngeal nerve.

During surgery, and after identification of all parathyroid glands, special care was taken to preserve the vascular pedicle of the parathyroid glands. None of our patients required damaged parathyroid glands autotransplanted into the sternocleidomastoid muscle.

## Results

Mean age of the patients was 44,5 years (range: 28–67). Gender ratio was 5.2 females for each 1 male (47/9).

**Figure 1:**
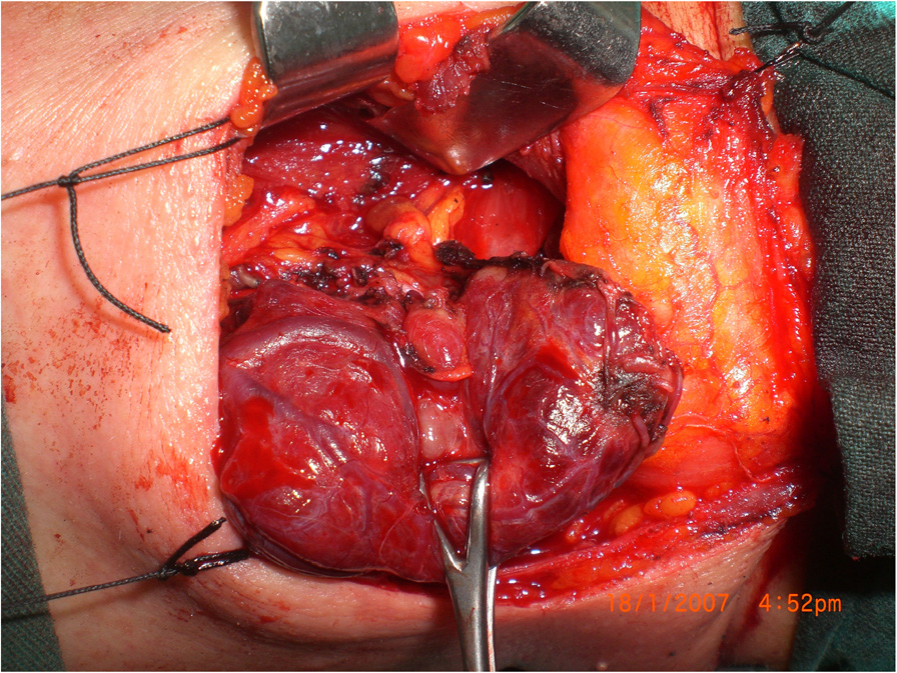
Sample 1, before methylene blue sprayed.

**Figure 2:**
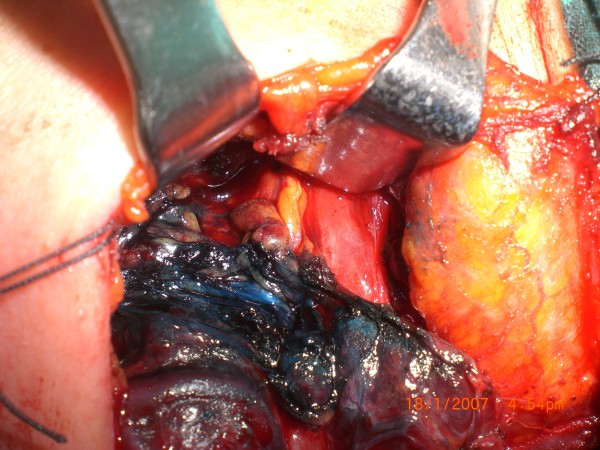
Sample 1, seconds after methylene blue sprayed (↓ Recurrent laryngeal nerve).

**Figure 3:**
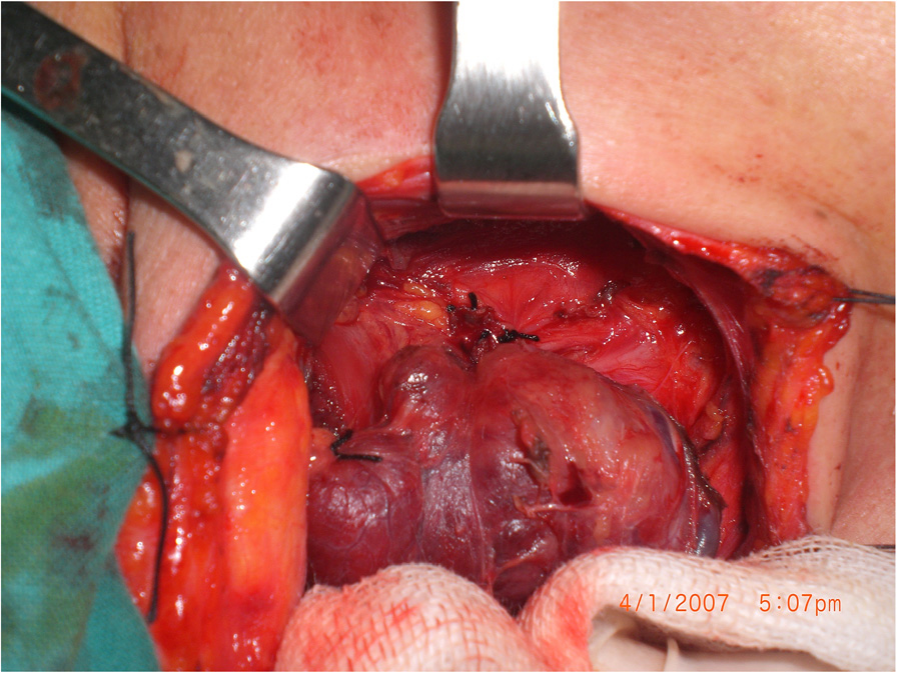
Sample 2, before methylene blue sprayed.

**Figure 4:**
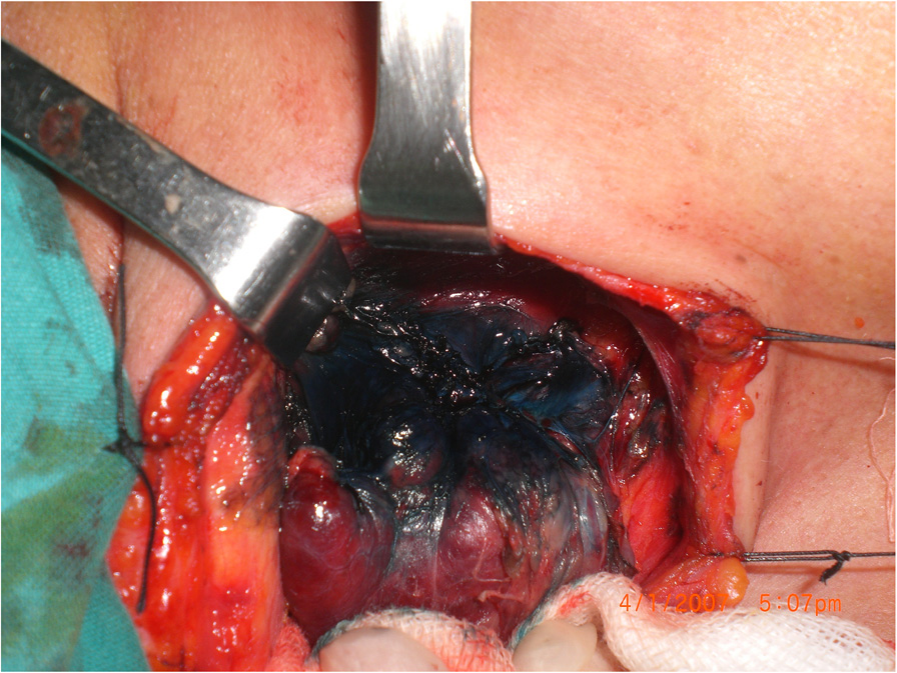
Sample 2, 30 seconds after methylene blue sprayed.

**Figure 5:**
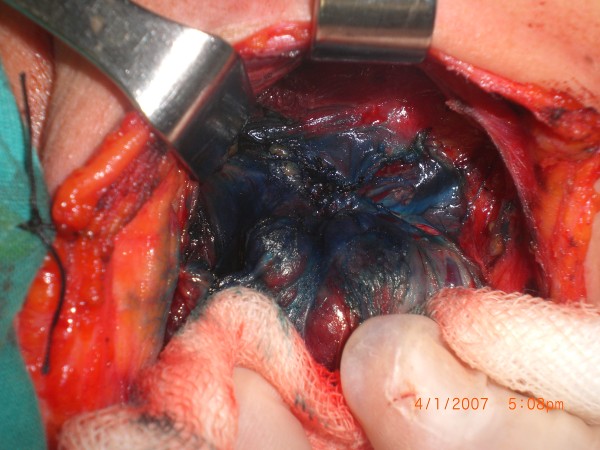
Sample-2, five minutes after methylene blue sprayed (? parathyroid glands).

The arteries and recurrent laryngeal nerves were not stained and therefore they remained white at all times (Figures 1, 2). Within three minutes, the parathyroid glands had washed out the blue stain so that the tissues were again their original yellow color (Figures 3, 4, 5). Wash-out time for thyroid tissue was not less than 15 minutes and not less than 25 minutes for perithyroideal muscles, tendinous, and lipoid structures. Staining and wash-out times of non-nodular thyroid lobes were diffuse in all cases. On the other hand, staining patterns of nodular thyroid lobes were not diffuse, but wash-out time was not shorter than 15 minutes. Patients were discharged in a mean of 1.2 days (1–3 days) with no major complications.

No operative mortality occurred and the incidence of transient hypoparathyroidism was a mere 5%. Only 3 cases had transient hypocalcemia, for a maximum of three days. Hypoparathyroidism improved with oral calcium supplementation with subsequent normal serum intact parathyroid hormone levels. Patients in our series encountered no persistent vocal cord paralysis or hypoparathyroidism

## Discussion

Morbidity and mortality from thyroidectomy occur rarely; however, possible serious postoperative complications could cause a devastating life-long handicap. Currently, the main postoperative complications of thyroidectomy are hypoparathyroidism and recurrent laryngeal nerve injury. The extent of resection, reoperation for completion, patient volume per surgeon and the surgeon’s inexperience are risk factors for morbidity of thyroid surgery. Meticulous dissection is a key factor in minimizing the development of complications [13-18].

Postoperative hypoparathyroidism is a major concern may lead to prolonged hospitalization and increased cost. In several studies, the incidence of transient hypoparathyroidism varied from 6.9% to 46% while a rate of 0.4% to 3.3% has been reported for permanent hypoparathyroidism. Falk et al. reported that transient hypoparathyroidism occurred in 27.8% of their cases manifested mostly as transient hypocalcemia, easily managed with oral supplementation of vitamin D and Ca^3^[19].

In the literature, all research related to thyroid surgery and staining of parathyroid glands was performed through intravenous and/or intra-arterial methylene blue injections. Dudley et al. used an intravenous infusion technique on 17 patients. In all cases, one or more of the parathyroids have been demonstrated with histological confirmation. Elias et al. used the same technique on 59 consecutive patients undergoing thyroid gland surgery (including 23 with carcinoma). Precise localization of the glands was possible in 87%. The intravascular (intravenous and/or intra-arterial) techniques described above ensure only parathyroid gland visualization and, accordingly, contributes to the prevention of hypoparathyroidism [20-24].

The other major complication in thyroid surgery [25] is recurrent laryngeal nerve palsy. This results in significant impairment of the quality of life [26] and negatively impacts on job performance [27-29]. Erbil et al. reported that recurrent laryngeal nerve palsy occurred in 1.8% of their cases [30].

To help identify the RLN and measure its function immediately before thyroid resection, various medical devices have been developed over the past two decades for intraoperative use. Several methods have been described for RLN monitoring including finger palpation of the cricoarytenoid muscle during nerve stimulation, vocal cord observation by direct or fiberotic laryngoscopy and the use of intramuscular vocal cord electrodes [31].

In a recent multicenter trial of 16,448 thyroidectomies, Dralle H. et al. concluded that visual nerve identification, in respect to RLN treatment, emerged as the “gold standard” of care [32].

Methylene blue is a hetero-cyclic aromatic chemical compound [33-38] which in recent years has been widely used in sentinel lymph node biopsies [39,40]. For prevention of hypoparathyroidism, staining of the parathyroid glands is not a new technique but was first described by Klopper et al. in 1966 [12]. Dyes first used by authors were toluidine blue and trypan blue [41,42], a derivative isomer of toluidine. After their potential teratogenic effects were discovered, they began to be replaced by methylene blue [43].

Studies in the literature show no significant differences between complication rates in their study cohorts and in patients undergoing total or subtotal thyroidectomy without the methylene blue spraying technique. Our research, on the other hand, shows major differences.

We did not use an expensive device for identifying the important structures. The dye is inexpensive, it can be easily and safely applied, unless by an inexperienced surgeon. We sprayed the dye onto the perithyroidal area, which is not normally used via the intravascular approach. Also, we aimed to identify not only parathyroid glands but also recurrent laryngeal nerve and inferior thyroid artery.

We observed that the wash-out time of parathyroid glands was less than three minutes but for thyroid glands was more than 15 minutes. We hypothesize that, the differences in time are due to the lympho-vascular pattern of the tissues. Histologically, the lympho-vascular structure of parathyroid glands is extremely dense. This peculiarity of the tissue is vital for immediate wash out of methylene blue staining. Unstaining of the recurrent laryngeal nerve during the procedure is not surprising because, like other peripheral nerves, it is covered by a schwann sheath and also has an avascular structure. Arterial non staining is due to its thick wall structure as well as reverse blood flow (not from tissue to heart but from heart to tissue) and. Because veins transport methylene dye from tissues, they immediately turn into blue in color.

Conventionally, surgeons identify the RLN by using judging its relationships with the inferior thyroid artery, tracheoesophageal groove, and ligament of Berry as anatomical landmarks. However, because of the numerous variations of this neurovascular relationship altered also by pathologic conditions of the gland, identification of the artery does not assure accurate identification and preservation of the recurrent laryngeal nerve. After spraying the dye onto the perithyroidal area, however, a surgeon can easily identify the recurrent laryngeal nerve, parathyroid glands and inferior thyroid artery. Once found, the nerve with all the identified branches can be quickly and safely followed through its entire course until it enters the larynx. When all the parathyroid glands have been identified, special care then can be easily taken to preserve their vascular pedicles.

Intraoperative stres can be increased by various intraoperative stressors, e.g., rising intolerance of physician error, potential legal issues, and medical insurers can increase. Kern et al. pointed out that surgical injuries accounted for the greatest number of lawsuits and the highest cost of litigation [44].

## Conclusion

During thyroidectomy most surgeons aim to preserve the nerves and parathyroid glands from potential risks. The sooner the nerve and parathyroid glands are identified, the lower the surgeon’s level of stress.

Our new technique for safe thyroid surgery is based on visualization of the parathyroid glands, recurrent laryngeal nerve, and thyroid arteries. We demonstrated the effectiveness of the spraying technique plus the lack of necessity of intravascular cannulation, along with its potential risks. This new technique ensures not only identification of parathyroid glands within three minutes, but also identification of the recurrent laryngeal nerve and thyroid arteries. New studies with larger numbers of cases and also application of the technique by different surgeons are important to confirm the reliability and effectiveness of this technique.

## Competing interests

The authors declare that they have no competing interests.

## Authors’ contribution

EA conceived of the study. EA and SS participated in the design of the study and drafted the manuscript. YE, HB and EY were contributor in writing the manuscript. MM participated in its design and coordination and helped to draft the manuscript. All authors read and approved the final manuscript.
